# Comparison of Canal Preparation Pattern of K3 and ProTaper Rotary Files in Curved Resin Blocks

**Published:** 2008-04-02

**Authors:** Nahid Mohammadzade Akhlaghi, Zohreh Khalilak, Ladan Baradaran Mohajeri, Mahshid Sheikholeslami, Saeed Saedi

**Affiliations:** 1 *Department of Endodontics, Dental School of Islamic Azad University and Member of Iranian Center for Endodontic Research, Tehran, Iran*; 2 *Department of Endodontics, Dental School of Islamic Azad University, Tehran, Iran*; 3 *General Practitioner, Tehran, Iran*

**Keywords:** K3, ProTaper, Root Canal Preparation, Root Canal Transportation, Rotary Files

## Abstract

**INTRODUCTION:** The purpose of this study was to evaluate and compare canal preparation pattern of K3 and ProTaper rotary files in curved resin blocks.

**MATERIALS AND METHODS:** Twenty-four resin blocks were used in this experimental study and randomly divided into two groups. Their initial images were scanned. After preparation, their images were scanned again in the same position. Pre and post preparation images were superimposed by Photoshop software and the removed resin was measured in 5 different points, and then analyzed statistically by ANOVA and t-test.

**RESULTS:** At O point (orifice), significantly (p<0.05) more outer canal wall was removed in the ProTaper group than in the K3 group. There was no significant difference at any other points of outer wall. Removed material of inner canal wall was not significantly different between the two groups.

**CONCLUSION:** Under the condition of this study, both systems performed acceptable preparation pattern except at the beginning of the curve.

## INTRODUCTION

Canal shaping is a critical phase of endodontic treatment because it influences the outcome of the subsequent phases of canal irrigation and filling and the success of the treatment itself. Once the canal is prepared, it should have a uniformly tapered funnel shape (1). The nature of canal dimensions, shape, and curves as well as the physical properties of instruments prevents the possibility of a uniform, tapered, flowing preparation (2). Canal shaping is relatively easy in straight roots but has always been challenging, demanding a high skill, when performed in curved roots (3). The quality guide line of the European Society of Endodontology states that the elimination of residual pulp tissue, the removal of debris and the maintenance of the original canal curvature during enlargement are the main objectives of root-canal instrumentation (4).

Many reports have described the tendency of root canal preparation techniques to cause canal transportation and other procedural problems such as ledging, apical perforation, and mid-root strip perforation. These complications may compromise the long-term success of treatment by failing to eliminate infection of the root canal system and making obturation more difficult. Various instrumentation techniques and instruments have been introduced in an attempt to reduce these problems aiming to provide the optimum shaped preparation (5).

The introduction of nickel titanium, or NiTi rotary instrumentation has made endodontics easier and faster than hand instrumentation, resulting in consistent and predictable root canal shaping (6).

The development of new design features such as varying tapers, non-cutting safety tips and varying length of cutting blades in combination with the metallurgic properties of NiTi alloy has resulted in a new generation of instruments (7).

The NiTi ProTaper file system (Dentsply, Maillefer, Ballaigues, Switzerland) is a relatively new endodontic rotary canal preparation technique. The manufacturer claims that these files are specially designed to instrument difficult highly calcified and curved root canals (8). The basic system is comprised of three shaping and three finishing instruments. The ProTaper files feature a triangular cross-section that reduces the contact area between the file and dentin, and provide what is described as a “minimally aggressive” cutting tip (8).

The K3 Endo NiTi rotary file system (Sybron Endo, Orange, USA) was introduced in 2002. These files are designed with a wide radial land, which is meant to make the instrument more resistant to torsional and rotary stresses. It also features “radial land relief”, which aids in protecting the file from “over engagement”, in the canal; thus, less instrument separation or distortion should occur. This file features a variable core diameter designed to increase flexibility, and it has a safe-ended tip to decrease the incidence of ledging, perforation, and zipping (9). Numerous studies have shown that Ni-Ti rotary instruments can effectively produce a well-tapered root canal form sufficient for obturation, with minimal risk of transporting the original canal (10-14).

Guelzow *et al. *compared various parameters of root canal preparation using a manual technique and six different rotary NiTi instruments (FlexMaster, System GT, HERO 642, K3, ProTaper, and RaCe). They concluded that all Ni-Ti systems maintained the canal curvature and were more rapid than a standardized manual technique. ProTaper instruments created more regular canal diameters (15). Veltri *et al. *analyzed the abilities of ProTaper and GT Rotary files to shape the curved canals of extracted mandibular molars (16). The dentin removal and the mean symmetry showed no significant differences between the two systems. Ankrum *et al*. investigated the incidence of file breakage and distortion when the ProTaper, K3 Endo and ProFile systems were used to instrument canals in the severely curved root canal of extracted molars (9). The results of their study showed that these three rotary tapered systems were not significantly different with regard to breakage. There were significantly more distorted files in the Profile group compared to the ProTaper group. With regard to distortion, there was no significant difference between the ProTper and K3 Endo and the ProFile and K3 Endo groups. Jodway *et al. *compared several parameters of curved root canal preparation using NiTi-TEE and K3 rotary NiTi instruments (17). Both systems maintained original canal curvature well and were safe to use. Whilst debridement of canals was considered satisfactory, both systems failed to remove smear layer sufficiently.

The purpose of this study was to compare the canal preparation pattern of K3 and ProTaper rotary files in curved resin blocks.

## MATERIALS AND METHODS

Twenty-four transparent resin simulated root canal blocks (Dentsply, Maillefer, Ballaigues, Switzerland) were used to assess instrument- tation. The degree of curvature was 45° and the radius of the curvature was 13 mm. They were randomly divided into two groups of 12 canals each.

Three landmarks were made with a round bur in the resin block from side wall to near inner and outer curve of the canal without penetrating into canal. These landmarks ensured a precise matching of pre and post operative images. Preoperative images of resin blocks in a fixed position were prepared using CanoScan 4200 F (Canon, Tokyo, Japan).

**Preparation of simulated canals**

Group 1- ProTaper Rotary System: ProTaper files (shaping and finishing files) were used in pecking motion as follows; size S1 (#17/variable taper) was advanced to resistance but no more than two third of the canal depth. The SX file was then introduced into the canal in a brushing action to 3-5 mm short of the working length. Then S1 and S2 were used at the working length. ProTaper finishing files F1 and F2 were used at the working length.

Group 2- K3 Rotary System: This group was prepared with K3 Rotary files (Sybron Endo, orange, California, USA) using VTVT technique. The K3 rotary system compromises 6 Ni-Ti files (two orifice shaper and 4 shaping files). Instruments were advanced apically in a gentle pecking motion until the first sign of resistance was detected. The following instruments were chosen to create a crown-down sequence (18-19):

Coronal Preparation:

#25: 0.10 taper: orifice shaper

#25: 0.08 taper: orifice shaper 1/3-2/3 of WL Crown-down to WL (Proceeding in 1 mm increments)

#35: 0.06

#30: 0.04

#25: 0.06: full WL

Canal preparation was completed with a master apical file of size 25 in all groups. Sodium hydrochloride (1%) was used for irrigation through a 31-gauge needle after use of each instrument. Each root canal was irrigated with a total of 30 mL sodium hydrochloride. The amount of RC Prep (Stone Pharmaceuticals, Philadelphia, PA) was enough to cover all the flute area of each file. Canal recapitulation was performed after the use of each file. Files were regularly wipes using wet gauze to remove resin debris. Patency and working length of each canal were determined by passing the 10 K-file (Dentsply, Maillefer, Ballaigues, Switzerland). All instrumentation was performed according to each manufacturer’s instructions (8-18). Two systems were used in crown-down technique with a hand-piece powered by an electric motor control (Endo-Mate DT motor, NSK, Tokyo, Japan).

To reduce interoperation variables each preparation was conducted by the same operator. One set of instruments were used for preparation of 4 canals.

Each block was then scanned in the previous fixed position. Superimposition of the pre and post operative specimens was aided by landmarks placed in the sides of the resin blocks. The superimposed pre and post-instrumentation stored images were analyzed using the Adobe Photoshop 8 software which magnified the canal images 10 times.

**Figure 1:**
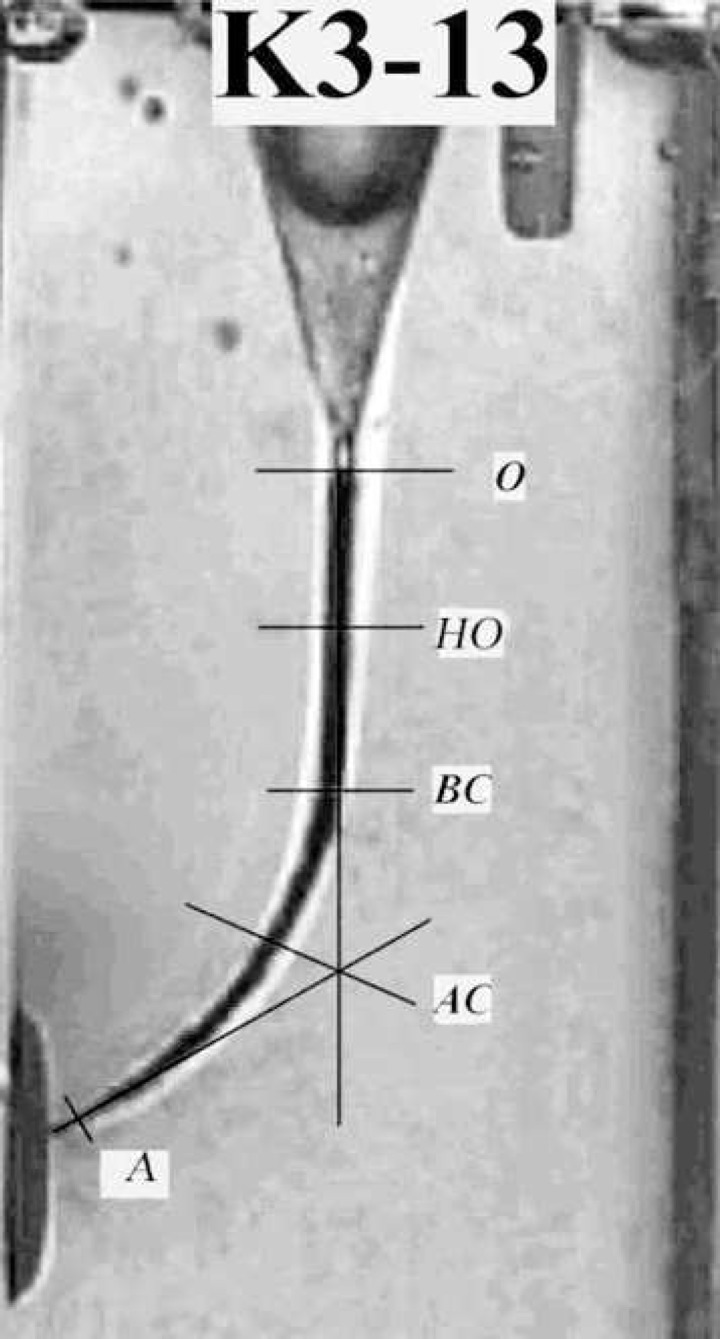
All measurements were made perpendicular to the axis of the pre-instrumentation canal using the image analysis software.

The removed resin were calculated at 5 different points at: canal orifice (O); half way to the orifice in the straight section (HO); the beginning of the curve (BC); the apex of the curve (AC); the end point (EP) (20) (Figure 1).

The increase in canal width due to the instrumentation process was recorded on both the inner and outer sides of the original canal.

Preparation time was recorded by using chronometer (accuracy 0.01 second) for both groups without the time for irrigation and changing the files.

**Recording, storage and analysis of data**

All data were recorded and stored in a PC. Following error and range checks, the data were analyzed using SPSS (SPSS Inc, Chicago, IL, USA), a statistical analysis program. Differences at the five points, between the mean total widths, mean inner wall widths and mean outer walls widths, in each group were statistically analyzed using t-test. These differences at the five points, between the two groups were statistically analyzed using ANOVA. A level of P<0.05 was considered significant.

## RESULTS

**Figure 2:**
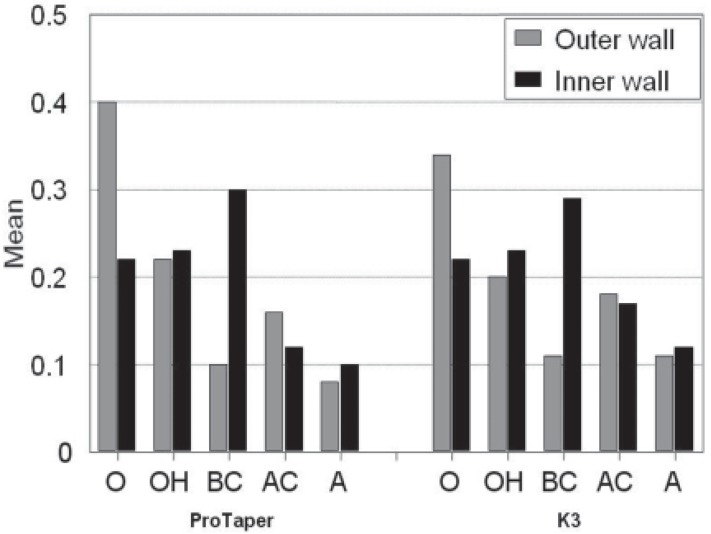
Comparison of material removal (mm) from outer and inner canal walls at different measuring points for each file

Resin removal amount at inner and outer canal walls is detailed in Figure 2.

***K3 group: ***In the K3 group, significantly more material was removed on the outer wall at O and on the inner wall at BC. There was no significant difference in the amount of material removed on the outer wall and inner wall in the other points.

**Figure 3:**
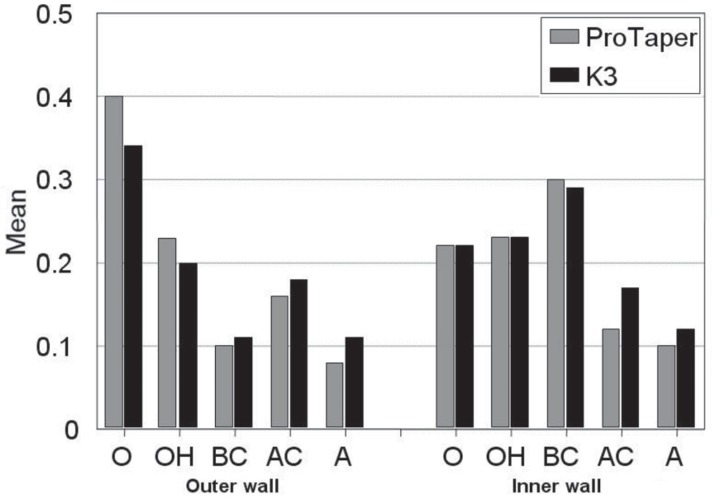
Comparison of material removal (mm) for two different files at different measuring points for inner and outer canal walls

***ProTaper group: ***More material was removed on the outer wall at O point. More resin was removed on the inner wall at BC point. There were significant differences in the amount of material removed on the outer and inner walls at O, BC, and A.

Figure 3 presents the result comparing the ProTaper and K3 groups and demonstrates that in the ProTaper group significantly (P<0.05) more outer canal wall was removed than in the K3 group at O point. There was no significant difference at any other points.

The amount of inner canal wall material removed was not significantly different between the two instruments (Figure 3).

K3 files were significantly faster (190.75±5.08 sec) than ProTaper file system (199.83±2.44 sec) (P<0.05).

## DISCUSSION

The purpose of this study was to compare the shaping ability of ProTaper with K3 files in simulated curved root canals.

The analysis of the canal width after instrumentation revealed that in the ProTaper group significantly (P<0.05) more outer canal wall was removed than in the K3 group, only at O point. At the other points, there were no differences between the two groups in the amount of material removed on the inner and outer walls.

In both systems more material was removed on the outer wall than inner wall at O point. This feature allows for ideal and efficient shaping of the coronal aspects of the root canal and the relocation of canal orifices, resulting in a straight line access. The relocation of the canal orifices should be in the direction of overhanging dentin areas and away from danger zones in furcation areas and thinner dentin walls, where strip perforations can compromise treatment objectives. At HO point, both systems removed more resin on the inner side of the curvature in the comparison with the outer side of the curvature. Although this differences was not statistically significant; but care should be taken with these instruments to avoid excessive removal at the inner curve, leading to straightening of the canal. Also, there was significantly (P<0.001) more resin removed on the inner wall than outer wall at BC for both systems, which resulted in straightening of the curved canals.

At AC point, both systems removed more resin from outer wall than inner wall. This difference was statistically significant in canals which prepared with ProTaper. Because of less dentin thickness on the inner wall in this area compared to outer wall, this pattern reduces the risk of stripping.

The results of this study revealed that K3 and ProTaper rotary systems removed more material from outer wall than inner wall, at the orifice. This is in agreement with Veltri *et al. *who reported that ProTaper instruments performed acceptable tapered preparation with minimal deviation from the original canal path (16).

In contrast with this study, Bergmans *et al. *(21) showed that ProTaper files removed more dentin from inner wall at coronal part than the other points and K3 files removed more dentin from the outer wall at apical point than the other points. These differences between these two studies might be because of different hardness and abrasion behavior of acrylic resin and root dentin. Yang *et al. *in their study showed that ProTaper instruments tended to transport towards the outer aspect of the L-shaped curved canals in the apical part and their results were in contrast with the present study (7).

In a recent study using simulated curved canals, ProTaper files had a higher risk of canal aberrations than GT Rotary, ProFile and RaCe (22). It has been shown that canal aberrations were produced following the use of the F2 and F3 instruments (20). Possibly less outer widening would have been created if preparation had been finished after F1 or F2.

Calberson *et al. *(20) showed ProTaper files removed more resin from the inner curve at the beginning of the curve, from the outer curve at orifice and equal resin from both walls at apical part. This pattern was similar to the present study. They used resin blocks with 40 degree curvature. Ayar and Love reported that the K3 instruments removed more resin on the outer wall than inner wall at O and AC, whilst at BC, the amount of resin removal on inner wall was more than the outer wall and at A, K3 instruments removed equal resin from outer and inner walls (5). These findings are almost in agreement with the present study and showed that K3 instruments prepared a well-shaped root canal with minimal canal transportation.

None of the K3 and ProTaper instruments fractured during preparation. It might be because of limited use of the files for preparation of canals (four canals). K3 instruments prepared canals significantly faster than ProTaper. This is in agreement with the findings of the study of Guelzow *et al. *(15).

## CONCLUSION

Under the conditions of this study, both rotary systems maintained original canal curvature well. However K3 instruments prepared canals faster than ProTaper.
